# Genome Sequence of *Actinobacillus seminis* Strain ATCC 15768, a Reference Strain of Ovine Pathogens That Causes Infections in Reproductive Organs

**DOI:** 10.1128/genomeA.01453-17

**Published:** 2018-01-11

**Authors:** Erasmo Negrete-Abascal, Fernando Montes-Garcia, Sergio Vaca-Pacheco, Abraham M. Leyto-Gil, Edgar Fragoso-Garcia, Roberto Carvente-Garcia, Sandra Perez-Agueros, Hugo G. Castelan-Sanchez, Alejandra Garcia-Molina, Tomas E. Villamar, Patricia Sánchez-Alonso, Candelario Vazquez-Cruz

**Affiliations:** a Carrera de Biología, Facultad de Estudios Superiores Iztacala, UNAM, Tlalnepantla, Estado de México, Mexico; b Centro Nacional de Referencia en Detección de Organismos Genéticamente Modificados, SENASICA, SAGARPA, Tecamac de Felipe Villanueva, Estado de México, Mexico; c Centro de Investigaciones en Ciencias Microbiológicas, Instituto de Ciencias, Benemérita Universidad Autónoma de Puebla, Puebla, Mexico

## Abstract

The draft genome sequence of *Actinobacillus seminis* strain ATCC 15768 is reported here. The genome comprises 22 contigs corresponding to 2.36 Mb with 40.7% G+C content and contains several genes related to virulence, including a putative RTX protein.

## GENOME ANNOUNCEMENT

*Actinobacillus seminis* is a Gram-negative bacterium, a member of the *Pasteurellaceae* family, and a causal agent of epididymitis and testicular lesions in sheep, producing low rates of fertility and abortions (1, 2) and consequent economic losses to the sheep industry (3). This bacterium is also known to affect other organisms, since it has been isolated from other ruminants (4). Pathogenicity and virulence factors expressed by this microorganism are mainly unknown (5–7), and this lack of awareness makes it difficult to control. To identify the pathogenic characteristics of the *A. seminis* genome, the ATCC 15768 strain was used for whole-genome sequencing.

Total genomic DNA of this bacterium was obtained from fresh cultures in brain heart infusion (BHI) medium. Cells were incubated with lysozyme and disrupted with sodium lauryl sarcosine. DNA was purified by phenolic extraction, RNase digestion, and sodium acetate precipitation. Purified high-molecular-weight DNA was processed at the Laboratorio del Centro Nacional de Referencia en Detección de Organismos Genéticamente Modificados, SENASICA, SAGARPA, to determine the nucleotide sequence. Following the instructions of the manufacturer, genomic libraries were prepared for DNA sequencing using a Roche 454-GS Junior next-generation sequencer (454 Life Sciences, Branford, CT, USA) (8). Reads were filtered based on quality, and cutting adapters were cleaned. Next, the reads were assembled with Genomics Workbench software (CLC bio), and 22 contigs with 100-fold coverage were obtained. The longest contig was 455,093 bp, and the shortest contig was 2,842 bp. The assembly produced an estimated genome of approximately 2.36 Mb, with 40.7% G+C content. An initial gene inspection was made with the Rapid Annotation using Subsystem Technology (RAST) pipeline (9). Finally, automatic gene prediction and annotation were performed using the NCBI Prokaryotic Genome Annotation Pipeline (https://www.ncbi.nlm.nih.gov/genome/annotation_prok/). With these tools, we identified 54 sequences related to the formation of functional RNAs and 33 related to prophages. Genes involved in heavy metal resistance, antimicrobial resistance, and oxidative stress were also identified, as were genes coding for a putative RTX protein.

### Accession number(s).

This whole-genome shotgun project has been deposited at DDB/EMBL/GenBank under the accession number NLFK00000000. The version described in this paper is the first version, NLFK01000000.
